# Enteroaggregative Escherichia coli.

**DOI:** 10.3201/eid0402.980212

**Published:** 1998

**Authors:** J P Nataro, T Steiner, R L Guerrant

**Affiliations:** University of Maryland School of Medicine, Baltimore 21201, USA. jnataro@umppa1.ab.umd.edu

## Abstract

Enteroaggregative Escherichia coli (EAEC), an increasingly recognized cause of diarrhea in children in developing countries, has been particularly associated with persistent diarrhea (more than 14 days), a major cause of illness and death. Recent outbreaks implicate EAEC as a cause of foodborne illness in industrialized countries. The pathogenesis of EAEC infection is not well understood, but a model can be proposed in which EAEC adhere to the intestinal mucosa and elaborate enterotoxins and cytotoxins, which result in secretory diarrhea and mucosal damage. EAEC's ability to stimulate the release of inflammatory mediators may also play a role in intestinal illness.


### Enteroaggregative *Escherichia coli*

**James P. Nataro,* Theodore Steiner,† and Richard L. Guerrant†**
*University of Maryland School of Medicine, Baltimore, Maryland, USA; and
†University of Virginia School of Medicine, Charlottesville, Virginia, USA

Enteroaggregative *Escherichia coli* (EAEC), an increasingly recognized cause of diarrhea in children in developing countries, has been particularly associated with persistent diarrhea (more than 14 days), a major cause of illness and death. Recent outbreaks implicate EAEC as a cause of foodborne illness in industrialized countries. The pathogenesis of EAEC infection is not well understood, but a model can be proposed in which EAEC adhere to the intestinal mucosa and elaborate enterotoxins and cytotoxins, which result in secretory diarrhea and mucosal damage. EAEC's ability to stimulate the release of inflammatory mediators may also play a role in intestinal illness.

Since first described in 1987, enteroaggregative *Escherichia coli* (EAEC) have been recognized increasingly as agents of diarrhea in developing countries and (more recently) in industrialized countries. We review the data supporting the pathogenicity of EAEC, discuss clinical and epidemiologic features, and summarize the current status of EAEC pathogenesis research.

### History

*E. coli* have been implicated as agents of diarrheal disease since the 1920s (1). In 1979, Cravioto et al. reported that most enteropathogenic *E. coli* (EPEC) adhered to HEp-2 cells in culture and that the adherence phenotype could be used to differentiate EPEC. Subsequently, many *E. coli* not of EPEC serogroups were also found to adhere to semiconfluent HEp-2 cells, but the adherence phenotype was clearly different from that induced by EPEC (2,3). Whereas the adherence pattern of EPEC was localized, the non-EPEC pattern was diffuse. The diffuse adherent strains were later subdivided into two further phenotypic subcategories: aggregative and true "diffuse" (4). Only the aggregative were associated with pediatric diarrhea in Santiago, Chile (4), prompting the investigators to propose two independent categories: EAEC and diffusely adherent *E. coli*. At the same time, non-EPEC, HEp-2 adherent strains (termed enteroadherent *E. coli*) were associated with pediatric and traveler's diarrhea in Mexico (5,6). These enteroadherent strains were later found to belong to the EAEC or diffusely adherent *E. coli* categories (7).

Thus, EAEC are now defined as *E. coli* that do not secrete heat-labile or heat-stable enterotoxins and adhere to HEp-2 cells in an aggregative (AA) pattern (Figure 1). This definition may encompass both pathogenic and nonpathogenic clones that share a factor(s) conferring a common phenotype.

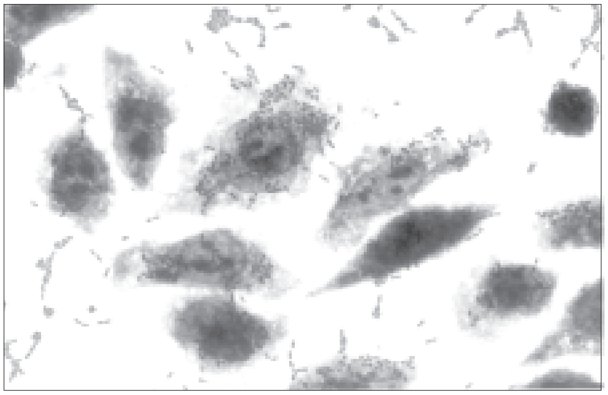

Figure 1. Aggregative pattern of adherence in the HEp-2 assay after 3 hours incubation of bacteria with HEp-2 cells. Note bacteria on the surface of the HEp-2 cells as well as on the glass substratum.

Address for correspondence: James P. Nataro, Center for Vaccine Development, University of Maryland School of Medicine, 685 W. Baltimore St., Baltimore, MD 21201, USA; fax: 410-706-6205; e-mail: jnataro@umppa1.ab.umd.edu.

### Pathogenesis

### Histopathology

EAEC strains adhere to the mucosal epithelium of gnotobiotic piglets in a thick mucus-containing blanket, irrespective of whether or not the strain induces diarrhea in this animal (8; Nataro, unpub. obs.; Figure 2). In these studies, the intestinal epithelium displayed pitting of goblet cells, suggesting stimulation of mucus secretion. EAEC strains adhered to sections of pediatric small bowel mucosa in an in vitro organ culture (IVOC) model (9); the bacteria were also embedded within a mucus-containing biofilm. Two further observations support a role of mucus in EAEC pathogenesis: EAEC bound rabbit mucus avidly in an in vitro model (10) and volunteers fed EAEC strains excrete mucoid stools (11). The formation of a heavy mucus biofilm may contribute to EAEC diarrheagenicity and, perhaps, to its ability to cause persistent colonization and diarrhea.

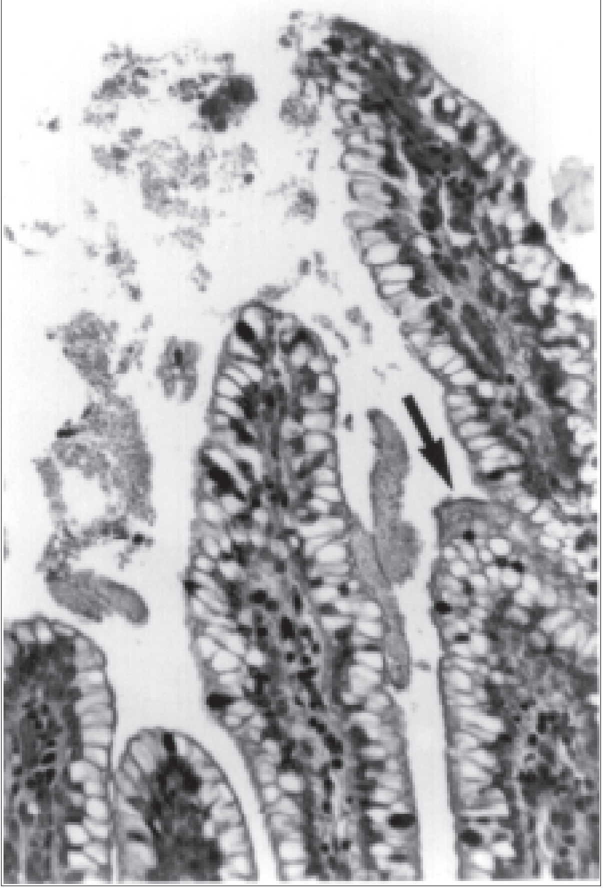

Figure 2. Biofilm (arrow), containing aggregating bacteria and mucus, adhering to the mucosa of a gnotobiotic piglet fed enteroaggregative *E. coli* strain 042 and sacrificed after 3 days. This piglet did not contract diarrhea. (Reprinted with permission of James Nataro and Clinical Microbiology Reviews. Clin Microbiol Rev 1998;11:142-201.)

In addition to forming the characteristic mucus biofilm, many EAEC strains induce cytotoxic effects on the intestinal mucosa. Infection with EAEC strains in rabbit and rat ileal loop models resulted in a destructive lesion demonstrable by light microscopy (7). EAEC induced shortening of the villi, hemorrhagic necrosis of the villous tips, and a mild inflammatory response with edema and mononuclear infiltration of the submucosa. Transmission electron microscopy showed normal microvillar architecture and no invasion of enterocytes; both light and electron microscopy showed adherent bacteria without the attaching and effacing lesion, which is characteristic of EPEC.

Mucosal damage has also been demonstrated in ileum specimens taken after patients died of EAEC persistent diarrhea during an outbreak in the malnutrition ward of Mexico City Hospital (12). This effect was reproduced in the IVOC model; EAEC induced exfoliation of enterocytes from the mucosal surface of pediatric intestinal biopsies (9). Indeed, EAEC strains elicit cytotoxic effects on T84 cells (human intestinal carcinoma) in vitro (13; Figure 3). In the in vitro model, EAEC induced the microvillar membrane to vesiculate and the cells from the monolayer to exfoliate. This effect was accompanied by increased vacuole formation and separation of the nucleus from the surrounding cytoplasm. Damage was most prominent in areas where EAEC were adhering to the cells. In both the T84 and IVOC systems, the toxic effects required genes encoding the adherence fimbriae (14), as well as other genes on the 65MDa plasmid.

### Adherence

Adherence of EAEC to the intestinal mucosa has been well studied. A flexible, bundle-forming EAEC fimbrial structure 2 nm to 3 nm in diameter, designated aggregative adherence fimbriae I (AAF/I) (15), mediates HEp-2 adherence and human erythrocyte hemagglutination in EAEC strain 17-2. The genes for AAF/I are organized as two separate gene clusters on the 60 MDa plasmid separated by 9 kb of intervening DNA (16-18). Region 1 genes encode the fimbrial structure itself; nucleotide sequence analysis of the Region 1 cluster suggests that

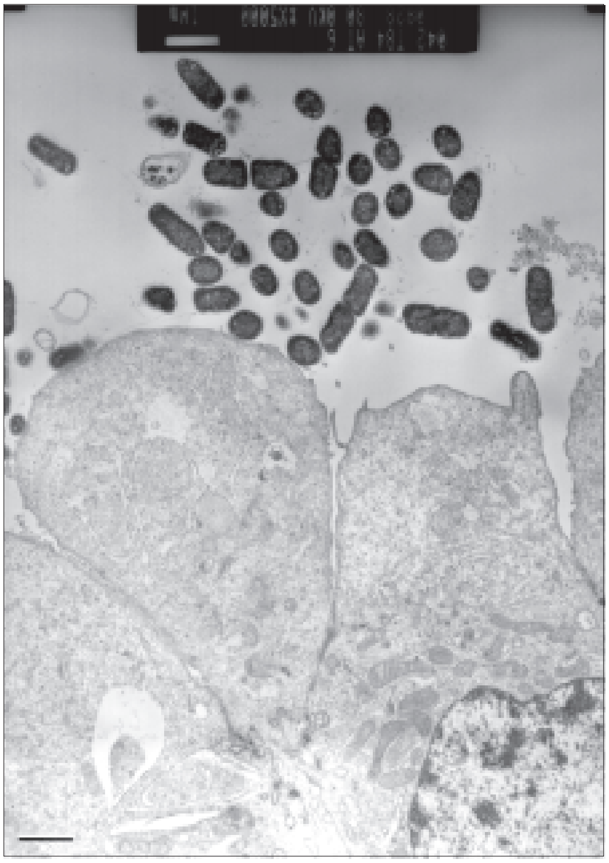

Figure 3. Effects of enteroaggregative *E. coli* strain 042 on T84 cells in culture. Polarized T84 monolayers were washed and inoculated with $10^6$ CFU of 042 and allowed to coincubate at 37˚C for 6 hrs. Transmission electron microscopy reveals adherence of bacteria to the apical surface of the T84 cells without internalization. The apical brush border is effaced; cells are ballooning and will eventually be extruded from the monolayer. (J.P. Nataro and S. Sears, unpub. data). Bar, 1 mm.

AAF/I is related to the Dr family of adhesins (18). Region 2 genes encode a transcriptional activator of AAF/I expression (AggR), which is a member of the AraC family of DNA binding proteins (17). The AAF/I fimbriae are bundle-forming fimbriae but do not show homology with members of the so-called type 4 class of fimbriae (19).

A second fimbrial structure, designated AAF/II, has been identified (14); it is distinct from but related to AAF/I. Insertional mutants in AAF/II genes no longer adhered to colonic mucosa in IVOC. DNA probe analysis suggested that AAF/I and AAF/II were each present in only a minority of EAEC strains and thus, as is the case with enterotoxigenic *E. coli*, intestinal colonization of EAEC appears to be mediated by one or more of several different fimbrial antigens.

In some EAEC strains, AA may be due to factors other than the AAF fimbriae (20,21). An afimbrial outer membrane protein or a 38 kDa outer membrane protein may be responsible for AA in some strains (20,21).

### EAEC ST-Like Toxin

Savarino et al. have identified an open reading frame encoding a 4,100 Da homologue of the heat-stable enterotoxin designated EAST1 (22,23), which is a 38-amino acid protein featuring four cysteine residues, instead of six (which are characteristic of *E. coli* ST). A role for EAST1 in human disease has not been demonstrated, although EAST1 clones yield net increases in short circuit current in the rabbit mucosal Ussing chamber model (22). Of the four strains given to volunteers, one that induced diarrhea, as well as one that did not, secreted EAST1 (11).

EAST1 has been found in approximately 40% of EAEC strains and in a similar proportion of nonpathogenic *E. coli* strains (24). Other *E. coli* categories, most notably EHEC, elaborate EAST1 with a higher frequency than EAEC (24).

### Invasiveness

Some EAEC strains may invade intestinal epithelial cells in vitro (25). However, human intestinal explants do not internalize EAEC, and clinical evidence for or against a role for invasiveness is lacking (9).

### Other EAEC Toxins

In an outbreak of EAEC diarrhea in Mexico, Eslava et al. identified (in serum from patients in the outbreak) an approximately 108 kDa protein that induces exfoliation of enterocytes in the rat ileal loop model. DNA sequence analysis of the plasmid-borne gene (C. Eslava, J. Czeczulin, F. Navarro-Garcia, I. Henderson, A. Cravioto, J.P. Nataro, unpub. obs.) encoding this protein suggests that the toxin is a member of the family of proteins (26) called autotransporters because their secretion through the bacterial outer membrane is mediated by the carboxy terminus of the molecule. The 108 kDa toxin (termed Pet for plasmid-encoded toxin) also induces enterotoxic activity from rat intestinal tissue mounted in the Ussing chamber (F. Navarro-Garcia, C. Eslava,

J.P. Nataro, A. Cravioto, unpub. obs.). A 120 kDa EAEC supernatant protein had been described that elicited rises in intracellular calcium in HEp-2 cells (27). Whether or not this protein is related to the protein described by Eslava et al. remains to be determined.

### Intestinal Inflammation

An ongoing study of infant diarrhea in Fortaleza, Brazil (28-30), has shown that children with EAEC infection have intestinal inflammation, as measured by the presence of inflammatory markers in the stool (31). These markers include lactoferrin, a stable neutrophil product and sensitive marker for fecal neutrophils; interleukin-8 (IL-8), a neutrophil chemokine; and interleukin-1β (IL-1β), a polyfunctional proinflammatory cytokine. Fecal lactoferrin, IL-8, and IL-1β were elevated in children with persistent diarrhea due to EAEC but not in controls (children in the same cohort without stool pathogens and free of diarrhea for 3 weeks before and after the time of the stool sample in prospective surveillance). Fecal lactoferrin and IL-1β were also elevated in children with EAEC but no diarrhea for 3 weeks. The origin of these inflammatory factors remains unknown, although they may be the result of direct stimulation of cytokine release from the epithelium.

While studying cytokine release in vitro, Steiner et al. observed IL-8 release from Caco-2 intestinal epithelial cells exposed to cell-free filtrates of EAEC strains 042 or 17-2. This release was apparently mediated by a heat-stable, high molecular weight, chromosomally encoded protein (31). Lipopolysaccharide does not appear responsible for this effect as it is not blocked by polymyxin B and is not elicited by normal *E. coli* flora.

The relationship between IL-8 release and the symptoms of EAEC infection is not known, especially since severe infiltration by neutrophils has not yet been reported in EAEC histopathologic specimens. However, a hypothetical role for IL-8 release in EAEC diarrhea can be envisioned. IL-8 released from epithelial cells in response to EAEC could act as the first step in a secretory cascade by recruiting neutrophils, since neutrophils in the intestinal epithelium release 5'-adenosine monophosphate, which is converted by a 5'-nucleotidase on the apical surface of enterocytes to adenosine, an agonist for chloride secretion (32). A similar mechanism of neutrophil chemotaxis and cytokine release may also be involved in the diarrhea produced by other microbial products, such as *Clostridium difficile* toxin A (33,34).

### Strain Heterogeneity

EAEC strains exhibit considerable heterogeneity. Studies at the University of Maryland found that at a dose of $10^{10}$ CFU with bicarbonate, strain 042 (which produces AAF/II fimbriae, EAST1, and the 108 kDa Pet toxin) elicited loose stools in 4 of the 5 adult volunteers (11). Three other strains, all of which expressed AAF/I and one of which secreted EAST1, did not produce any intestinal symptoms. These data suggest that virulence is likely to be heterogeneous and that Pet may play an important role.

### A Model of EAEC Pathogenesis

Extrapolating from the observations described above, we propose a three-stage model of EAEC pathogenesis. Stage I involves initial adherence to the intestinal mucosa and the mucus layer; this initial adherence is apparently mediated by the AAF fimbriae. Stage II comprises enhanced mucus production, apparently leading to the deposit of a thick mucus-containing biofilm encrusted with EAEC. The blanket may promote persistent colonization and nutrient malabsorption. Stage III, suggested from histopathologic and molecular evidence, includes the elaboration of toxins or inflammation, which result in damage to the mucosa and intestinal secretion. Malnourished hosts may be unable to repair the mucosal damage and may thus become prone to the persistent diarrhea syndrome.

The site of EAEC infection in the human intestine has yet to be clearly demonstrated. IVOC experiments have shown that EAEC strains can adhere to both small and large bowel mucosa (9). Tissue specimens in these studies were derived from pediatric patients and were not formalin-fixed before they were incubated with EAEC strains. These features may explain the discrepant results obtained by other investigators (35,36). The short incubation period observed in some humans challenged with strain 042 (as short as 8 hours) is also consistent with involvement of the small bowel in diarrheagenicity (11).

### Clinical Features

The clinical features of EAEC diarrhea are increasingly well defined in outbreaks, sporadic cases, and the volunteer model. Typical illness is manifested by a watery, mucoid, secretory diarrhea with low grade fever and little to no vomiting (37,38). However, up to one-third of patients with EAEC diarrhea have had grossly bloody stools (39); this outcome may be strain dependent. In volunteers infected with EAEC strain 042, the diarrhea was mucoid, of low volume, and without occult blood or fecal leukocytes; all patients remained afebrile. In such volunteers, the incubation period of the illness was 8 to 18 hours (11).

The duration of EAEC diarrhea is its most striking feature. In a community surveillance study in Anapur-Palla in northern India, the mean duration of EAEC-associated diarrhea cases in children under 3 years of age was 17 days, longer than that associated with any other pathogen (37). In this study, of the 41 cases of diarrhea, fever was found in 12%, gross blood in 12%, vomiting in 7%, and persistence of the episode more than 14 days in 44%.

A large percentage of patients excreting EAEC have detectable fecal lactoferrin and supranormal levels of IL-8 in the stool (31). Although this observation suggests that EAEC infection may be accompanied by mucosal inflammation, most patients lack overt clinical evidence of inflammation.

### Diagnosis

EAEC colonization is detected by isolating *E. coli* from stool samples and demonstrating the AA pattern in the HEp-2 assay. Analysis of small bowel aspirates has not increased yield (40). Implication of EAEC as the cause of the patient's disease must be done cautiously, given the high rate of asymptomatic colonization in many populations (4,30,41-43). If no other organism is implicated in the patient's illness and EAEC is isolated repeatedly, it may be considered to be a probable cause of the patient's illness.

Despite various methods of performing the HEp-2 assay (4,44,45), comparative studies suggest that the technique first described by Cravioto et al. (45,46) (i.e., a single 3-hour incubation of bacteria with cells, without a change in medium during the assay) best discriminates among the three adherence patterns. Because AAF adhesins are expressed maximally in static L-broth cultures at 37°C (15), we incubate all HEp-2 assay inocula in this manner.

A DNA fragment probe has proven highly specific in detecting EAEC strains. This probe was developed by Baudry et al. (47), who tested fragments derived empirically from the plasmids of strains 17-2 and 042. A 1.0 kb plasmid-derived *Sau*3a fragment hybridized with 56 (89%) of 63 EAEC strains (defined by HEp-2 assay); of 376 strains representing normal flora and other diarrheagenic categories, only 2 hybridized with the probe. The correlation of the EAEC probe-positivity with AA varies by location. In some studies, the correlation achieves the 89% sensitivity reported by Baudry et al. (47,48), while in other studies, the sensitivity may be substantially lower (40). The epidemiologic significance of probe-positive versus probe-negative strains has not been determined. The nucleotide sequence of the AA probe represents a cryptic open reading frame adjacent to the plasmid replicon (J.P. Nataro, unpub. obs.). A polymerase chain reaction assay using primers derived from the AA probe sequence shows similar sensitivity and specificity (49).

### Epidemiology

Volunteer studies and outbreak investigations have shown that at least some EAEC strains are human pathogens. A growing number of studies have supported the association of EAEC with diarrhea in developing countries; the increasing number of such reports and the rising proportion of diarrheal cases in which EAEC are implicated suggest that EAEC are important emerging agents of pediatric diarrhea.

### EAEC in Sporadic Diarrhea

The earliest epidemiologic reports showed that EAEC were most prominently associated with persistent (lasting ≥14 days) cases of pediatric diarrhea (Table 1). In some of these studies, EAEC cultured from the stool during the first few days of diarrhea were predictive of a longer illness (37,43).

The importance of EAEC in diarrheal disease appears to vary geographically. On the Indian subcontinent, six studies (which included hospitalized patients with persistent diarrhea [50], outpatients visiting health clinics [51], and cases of sporadic diarrhea detected on household surveillance [37]) have demonstrated the importance of EAEC in pediatric diarrhea (37,38,48,50,51,53).

Table 1. Epidemiologic studies associating enteroaggregative *Escherichia coli* (EAEC) with diarrhea ( $p < 0.05$)

| Source or reference | Syndrome | Site | EAEC in cases vs controls | Comments |
|---|---|---|---|---|
| 4 | All diarrhea | Santiago, Chile | 84(33%)/253 vs 20(15%)/134[a] | Community and hospital-based. First description of EAEC |
| 37 | Persistent diarrhea | Anapur-Palla, India | 18(30%)/61 vs. 20(10%)/201[b] | Household surveillance of rural children ≤3 yrs; first association of EAEC with PD |
| 50 | Persistent diarrhea | New Delhi, India | 18(20%)/92 vs 6(7%)/92[b] | Case-control study of nonbloody PD in children ≤ 2 yrs |
| 28 | Persistent diarrhea | Fortaleza, Brazil | 8(20%)/40 vs. 2(5%)/38[b] | Household surveillance of children ≤ 5 yrs |
| 39 | All diarrhea; persistent diarrhea | Morales, Mexico | 78(12%)/636[a] and 29(51%)/57[b] vs 5(5%)/100 | Household surveillance of children ≤ 2 yrs |
| 43 | Persistent diarrhea | Mirzapoor, Bangladesh | 17(27%)/62 vs 5(18%)/28[c] | Household surveillance of children ≤ 6 yrs. EAEC isolated in first 2 days of episode predicts persistence |
| 29 | Persistent diarrhea | Fortaleza, Brazil | 8(20%)/40 vs 2(5%)/38[b] | Outpatients ≤ 29 months with PD |
| 51 | All diarrhea | New Delhi, India | 9(21%%)/42 vs 4(4%)/107[b] | Children ≤ 4 yrs referred to hospital |
| 38 | Acute diarrhea | Calcutta, India | 17(11%)/159 vs 3(2%)/174[d] | EAEC associated with acute secretory diarrhea in hospitalized pediatric patients |
| 42 | All diarrhea | Tehran, Iran | 99(32%)/309 vs 17(17%)/100[a] | Pediatric outpatients. High rate of antibiotic resistance among EAEC |
| 39 | Persistent diarrhea | Fortaleza, Brazil | 38(68%)/56 vs 13(31%)/42[b] | Outpatients ≤ 3 yrs; EAEC associated with more cases of PD than all other agents combined |
| 48 | Acute diarrhea | Vellore, India | 60(8%)/794 vs 22(4%)/566[a] | Outpatients ≤ 3 yrs with diarrhea ≤ 3 days |
| 52 | All diarrhea | Wurzberg, Germany | 16(2%)/798 vs 0/580[a] | Hospitalized children ≤ 16 yrs. First description of EAEC disease burden in industrialized countries |
| 41 | Acute diarrhea | Caracas, Venezuela | 138(27%)/513 vs 36(15%)/241[a] | Inpatient and outpatient children ≤ 2 yrs with diarrhea ≤ 3 days. EAEC only associated with diarrhea in infants ≤ 2 mos of age |
| Jalla-luddin et al., 1997, pers. comm. | All diarrhea | Lwiro, Zaire | 29(25%)/115 vs 3(8%)/34[a] | Outpatients ≤ 5 yrs. First description of EAEC in Africa |

[a]All diarrhea vs. asymptomatic.
[b]Persistent diarrhea (PD) vs. asymptomatic.
[c]Persistent diarrhea vs. acute diarrhea.
[d]Secretory vs. invasive diarrhea.

EAEC and persistent diarrhea syndrome have been consistently associated (28,29,30,40). In Brazil, EAEC have been implicated in up to 68% of persistent diarrhea cases (40), which represent a disproportionate share of deaths due to diarrhea. EAEC have also been implicated as causes of sporadic diarrhea in Mexico, Chile, Bangladesh, and Iran (4,39,42,43).

The epidemiologic characteristics of EAEC (e.g., likely sources, reservoirs of infection, routes of transmission, seasonality, and age-distribution) are largely unknown. Most studies in which EAEC are implicated as causes of diarrhea have isolated the organism from infants and small children, yet volunteer studies and outbreak investigations suggest that school-age children and adults are also susceptible (54-56). Regarding sources of infection, EAEC strains have been isolated from the lacteal contents of infant feeding bottles in Brazil (57).

Recent studies have suggested that EAEC are found frequently in stool samples of patients in industrialized countries and may be important agents of diarrhea. EAEC were isolated from 2% of pediatric patients with diarrhea in Germany; the pathogen was associated with diarrheal illness (58). The patients from whom EAEC were isolated had characteristically prolonged illness, often followed by apparent abdominal pain mimicking infant colic.

### EAEC Outbreaks

Several outbreaks of EAEC diarrhea have now been reported (Table 2). The first detailed description of an outbreak from which EAEC were clearly implicated involved 19 infants in a hospital nursery in Nis, Serbia, during 9 days in 1995 (52). Twelve of the 19 infants had the same multidrug-resistant EAEC strain of serogroup O4, while none of five well neonates had this organism ($p = 0.02$). The illness lasted 3 to 9 days (mean 5.2 days) in 16 babies, but in three infants, persistent diarrhea developed, lasting 18 to 20 days. Infants with diarrhea typically had liquid green stools. In three infants, mucus was visibly apparent; no infant had bloody stools. All but three infants required intravenous hydration but all survived. The source of infection was unclear.

In two outbreaks of severe lethal diarrhea in malnutrition wards of Mexico City hospitals (12), persistent diarrhea developed in affected infants, and five patients died despite aggressive support. Autopsy findings from infants who died in these outbreaks showed severe necrotic lesions of the ileal mucosa.

During a diarrhea epidemic in a village in southern India in January 1996, EAEC were identified in the stools of 11 of 20 persons who were thought to have outbreak-related diarrhea; 1 of 11 stools from asymptomatic controls from the same village ($p < 0.02$) contained EAEC (59). Intake of water from open wells was associated with diarrheal illness; however, EAEC were not identified from this source, and contaminated water was not definitively implicated as the source of the epidemic.

EAEC outbreaks also occur in industrialized countries. In a massive outbreak of EAEC diarrhea in Gifu Prefecture, Japan, in 1993, 2,697 children at 16 schools became ill after consuming contaminated school lunches (54). The illness was characterized by abdominal pain, nausea, and severe diarrhea, which was protracted in at least 30 cases. The incubation period for this illness averaged 40 to 50 hours. A single EAEC strain of serotype O:H10 was implicated, but the organism was not found in any of the foods served in the implicated lunch.

Four outbreaks of EAEC-related diarrhea occurred in the United Kingdom in 1994 (56), which involved 19, 10, 51, and 53 persons, respectively, most of them adults. Only in the last

Table 2. Outbreaks of enteroaggregative *Escherichia coli* diarrhea

| Reference | Setting | Patients | Illness |
|---|---|---|---|
| 52 | Nursery; Nis, Serbia, 1995 | 19 infants | Watery diarrhea |
| 56 | Conference Center, UK, 1994 | 51 adults | Watery diarrhea for 3-7 days |
| 56 | Hotel, UK, 1994 | 53 adults | Watery diarrhea, mean duration 68 hr |
| 59 | Village in southern India, 1996 | 20 children and adults | Watery diarrhea, mean duration 11 days |
| 12 | Nursery, Mexico City | Not reported | Severe diarrhea; 5 deaths |
| 54 | 16 schools, Gifu, Japan | 2,697 school children | Diarrhea; some cases protracted |

two outbreaks was a single EAEC strain implicated. Although each of these outbreaks was linked to consumption of a meal, no single vehicle has been implicated. The illnesses were characterized by vomiting and diarrhea, occasionally with fever. The mean duration of illness in one outbreak was 68 hours. Strains of *E. coli* O44:H18 implicated in outbreaks in the United Kingdom (and previously presumed to be EPEC) are in fact EAEC (55). This report mentions three EAEC outbreaks, two among children and one among adult patients.

### EAEC and Malnutrition

Perhaps even more significant than the association of EAEC with diarrhea may be the recent data from Fortaleza, Brazil, that link EAEC with growth retardation in infants (31). In this study, EAEC isolated from the stools of infants was associated with a low *z*-score for height or weight, irrespective of the presence of diarrheal symptoms. Such an association has been suggested for *Cryptosporidium* (60). A study among an Australian aborigine population supports the association of EAEC with growth retardation in the absence of diarrhea (S. Elliott, J.P. Nataro, unpub. data). Given the high prevalence of asymptomatic EAEC excretion in many areas (4,30,41-43), if this observation holds up in further studies, the contribution of EAEC to human disease might be much greater than is currently thought.

### Conclusions

EAEC is an emerging diarrheal pathogen linked to acute and persistent diarrhea in both developing and industrialized countries. Both sporadic diarrhea and outbreaks (possibly foodborne) have been described. The mechanisms of pathogenesis are being elucidated; likely not all EAEC strains are equally pathogenic. The mechanism of the association between EAEC and malnutrition is not entirely clear, although plausible pathogenetic models can be proposed. Malnutrition predisposes to persistent diarrhea (61,62); whether this is due to impaired host immunity (preventing normal killing of pathogens) or to altered intestinal physiology (predisposing to more severe infections) is not known. However, it seems also plausible that asymptomatic colonization with EAEC may lead to malnutrition caused by increased metabolic demand secondary to intestinal inflammation, persistent mucosal damage due to cytotoxins, and/or the presence of a barrier to the absorption of nutrients imposed by the mucus/bacteria biofilm. A vicious cycle may thus operate wherein malnutrition and infection perpetuate and enable each other (Figure 4). The pathophysiologic machinery that propels children through this circle is probably multifactorial and opens several possible avenues for intervention against this important global problem.

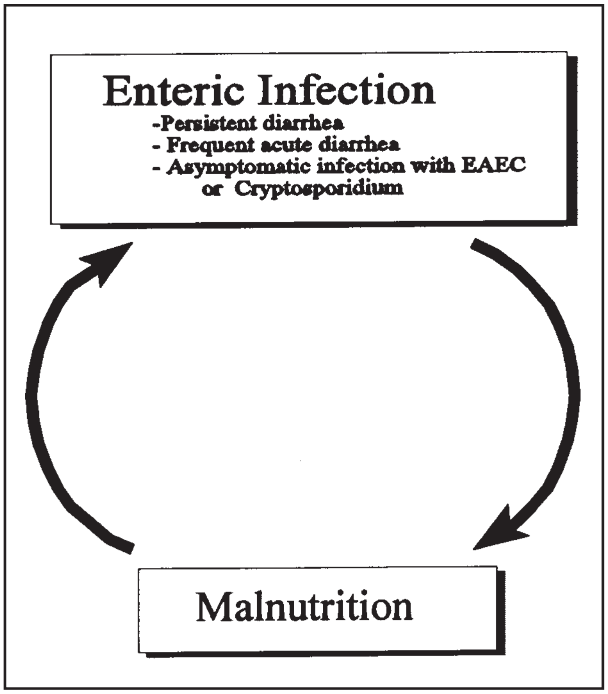

Figure 4. Relationship between diarrhea and malnutrition (31, 60–62).

Supported by Public Health Service grant AI 33096 to JPN.

Dr. Nataro is associate professor of pediatrics, medicine, and microbiology and immunology at the University of Maryland School of Medicine, Baltimore. His research focuses on the pathogenesis and epidemiology of the various pathotypes of diarrheagenic *Escherichia coli*, with particular interest in their role in persistent diarrhea among children in developing countries.

### References

1. Lior H. Classification of *Escherichia coli*. In: Gyles CL, editor. *Escherichia coli* in domestic animals and humans. Wallingford, United Kingdom: CAB International 1996:31-72.

2. Scaletsky ICA, Silva MLM, Trabulsi LR. Distinctive patterns of adherence of enteropathogenic *Escherichia coli* to HeLa cells. Infect Immun 1984;45:534-6.
3. Nataro JP, Scaletsky IC, Kaper JB, Levine MM, Trabulsi LR. Plasmid-mediated factors conferring diffuse and localized adherence of enteropathogenic *Escherichia coli*. Infect Immun 1985;48:378-83.
4. Nataro JP, Kaper JB, Robins Browne R, Prado V, Vial P. Levine MM. Patterns of adherence of diarrheagenic *Escherichia coli* to HEp-2 cells. Pediatr Infect Dis J 1987;6:829-31.
5. Mathewson JJ, Johnson PC, DuPont HL, Morgan DR, Thornton SA, Wood LV, et al. A newly recognized cause of traveler's diarrhea: enteroadherent *Escherichia coli*. J Infect Dis 1985;151:471-5.
6. Mathewson JJ, Johnson PC, DuPont HL. Pathogenicity of enteroadherent *Escherichia coli* in adult volunteers. J Infect Dis 1986;154:524-7.
7. Vial PA, Robins Browne R, Lior H, Prado V, Kaper JB, Nataro JP, et al. Characterization of enteroadherent-aggregative *Escherichia coli*, a putative agent of diarrheal disease. J Infect Dis 1988;158:70-9.
8. Tzipori S, Montanaro J, Robins-Browne RM, Vial P, Gibson R, Levine MM. Studies with enteroaggregative *Escherichia coli* in the gnotobiotic piglet gastroenteritis model. Infect Immun 1992;60:5302-6.
9. Hicks S, Candy DCA, Phillips AD. Adhesion of enteroaggregative *Escherichia coli* to pediatric intestinal mucosa in vitro. Infect Immun 1996;64:4751-60.
10. Wanke CA, Cronan S, Goss C, Chadee K, Guerrant RL. Characterization of binding of *Escherichia coli* strains which are enteropathogens to small-bowel mucin. Infect Immun 1990;58:794-800.
11. Nataro JP, Yikang D, Cookson S, Cravioto A, Savarino SJ, Guers LD, et al. Heterogeneity of enteroaggregative *Escherichia coli* virulence demonstrated in volunteers. J Infect Dis 1995;171:465-8.
12. Eslava C, Villaseca J, Morales R, Navarro A, Cravioto A. Identification of a protein with toxigenic activity produced by enteroaggregative *Escherichia coli* [Abstract]. Abstracts of the General Meeting of the American Society for Microbiology; 1993. Washington: The Society; 1993. Abstract B105:44.
13. Nataro JP, Hicks S, Phillips AD, Vial PA, Sears CL. T84 cells in culture as a model for enteroaggregative *Escherichia coli* pathogenesis. Infect Immun 1996;64:4761-8.
14. Czeczulin JR, Balepur S, Hicks S, Phillips A, Hall R, Kothary MH, et al. Aggregative Adherence Fimbria II, a second fimbrial antigen mediating aggregative adherence in enteroaggregative *Escherichia coli*. Infect Immun 1997;65:4135-45.
15. Nataro JP, Deng Y, Maneval DR, German AL, Martin WC, Levine MM. Aggregative adherence fimbriae I of enteroaggregative *Escherichia coli* mediate adherence to HEp-2 cells and hemagglutination of human erythrocytes. Infect Immun 1992;60:2297-304.
16. Nataro JP, Yikang D, Giron JA, Savarino SJ, Kothary MH, Hall R. Aggregative adherence fimbria I expression in enteroaggregative *Escherichia coli* requires two unlinked plasmid regions. Infect Immun 1993;61:1126-31.
17. Nataro JP, Yikang D, Yingkang D, Walker K. AggR, a transcriptional activator of Aggregative Adherence Fimbria I expression in enteroaggregative *Escherichia coli*. J Bacteriol 1994;176:4691-9.
18. Savarino SJ, Fox P, Yikang D, Nataro JP. Identification and characterization of a gene cluster mediating enteroaggregative *Escherichia coli* aggregative adherence fimbria I biogenesis. J Bacteriol 1994;176:4949-57.
19. Tennant JM, Mattick JS. Type 4 Fimbriae. In: Klemm P, editor. Fimbriae: Adhesion, Genetics, Biogenesis and Vaccines. Boca Raton (FL): CRC Press; 1994. p. 127-46.
20. Wai SN, Takade A, Amako K. The hydrophobic surface protein layer of enteroaggregative *Escherichia coli* strains. FEMS Microbiol Lett 1996;135:17-22.
21. Debroy C, Yealy J, Wilson RA, Bhan MK, Kumar R. Antibodies raised against the outer membrane protein interrupt adherence of enteroaggregative *Escherichia coli*. Infect Immun 1995;63:2873-9.
22. Savarino SJ, Fasano A, Robertson DC, Levine MM. Enteroaggregative *Escherichia coli* elaborate a heat-stable enterotoxin demonstrable in an in vitro rabbit intestinal model. J Clin Invest 1991;87:1450-5.
23. Savarino SJ, Fasano A, Watson J, Martin BM, Levine MM, Guandalini S, et al. Enteroaggregative *Escherichia coli* heat-stable enterotoxin 1 represents another subfamily of E. coli heat-stable toxin. Proc Natl Acad Sci U S A 1993;90:3093-7.
24. Savarino SJ, McVeigh A, Watson J, Cravioto A, Molina J, Echeverria P, et al. Enteroaggregative *Escherichia coli* heat-stable enterotoxin is not restricted to enteroaggregative *E. coli*. J Infect Dis 1996;173:1019-22.
25. Benjamin P, Federman M, Wanke CA. Characterization of an invasive phenotype associated with enteroaggregative *Escherichia coli*. Infect Immun 1995;63:3417-21.
26. Jose J, Jahnig F, Meyer TF. Common structural features of IgA1 protease-like outer membrane protein autotransporters. Mol Microbiol 1995;18:377-82.
27. Baldwin TJ, Knutton S, Sellers L, Hernandez HAM, Aitken A, Williams PH. Enteroaggregative *Escherichia coli* strains secrete a heat-labile toxin antigenically related to *E. coli* hemolysin. Infect Immun 1992;60:2092-5.
28. Wanke CA, Schorling JB, Barrett LJ, Desouza MA, Guerrant RL. Potential role of adherence traits of *Escherichia coli* in persistent diarrhea in an urban Brazilian slum. Pediatr Infect Dis J 1991;10:746-51.
29. Lima AAM, Fang G, Schorling JB, de Albuquerque L, McAuliffe JF, Mota S, et al. Persistent diarrhea in Northeast Brazil: Etiologies and interactions with malnutrition. Acta Paediatr Suppl 1992;381:39-44.
30. Fang GD, Lima AM, Martin CC, Barrett LJ, Nataro JP, Guerrant RL. Aggregative HEp-2 cell adherent *Escherichia coli* and Cryptosporidium: important pathogens in hospitalized children with persistent diarrhea in Northeast Brazil [Abstract]. Abstracts of the 32nd Interscience Conference on Antimicrobial Agents & Chemotherapy; 1992. Washington: American Society for Microbiology; 1997. Abstr. 686.
31. Steiner TS, Lima AAM, Nataro JP, Guerrant RL. Enteroaggregative *Escherichia coli* produce intestinal inflammation and growth impairment and cause interleukin-8 release from intestinal epithelial cells. J Infect Dis 1998;177:88-96.

32. Madara JL, Patapoff TW, Gillece-Castro B, Colgan SP, Parkos CA, Delp C, et al. 5'-adenosine monophosphate is the neutrophil-derived paracrine factor that elicits chloride secretion from T84 intestinal epithelial cell monolayers. J Clin Invest 1993;91:2320-5.
33. Rocha MFG, Maia MET, Bezerra L. *Clostridium difficile* toxin A induces the release of neutrophil chemotactic factors from rat peritoneal macrophages: role of interleukin-1', tumor necrosis factor alpha, and leukotrienes. Infect Immun 1997;65:2740-6.
34. Mahida YR, Makh S, Hyde S. Effect of *Clostridium difficile* toxin A on human intestinal epithelial cells: induction of interleukin 8 production and apoptosis after cell detachment. Gut 1996;38:337-47.
35. Knutton S, Shaw RK, Bhan MK, Smith HR, McConnell MM, Cheasty T, et al. Ability of enteroaggregative *Escherichia coli* strains to adhere in vitro to human intestinal mucosa. Infect Immun 1992;60:2083-91.
36. Yamamoto T, Echeverria P, Yokota T. Drug resistance and adherence to human intestines of enteroaggregative *Escherichia coli*. J Infect Dis 1992;165:744-9.
37. Bhan MK, Raj P, Levine MM, Kaper JB, Bhandari N, Srivastava R, et al. Enteroaggregative *Escherichia coli* associated with persistent diarrhea in a cohort of rural children in India. J Infect Dis 1989;159:1061-4.
38. Paul M, Tsukamoto T, Ghosh AR, Bhattacharya SK, Manna B, Chakrabarti S, et al. The significance of enteroaggregative *Escherichia coli* in the etiology of hospitalized diarrhoea in Calcutta, India and the demonstration of a new honey-combed pattern of aggregative adherence. FEMS Microbiol Lett 1994;117:319-26.
39. Cravioto A, Tello A, Navarro A, Ruiz J, Villafan H, Uribe F, et al. Association of *Escherichia coli* HEp-2 adherence patterns with type and duration of diarrhoea. Lancet 1991;337:262-4.
40. Fang GD, Lima AAM, Martins CV, Nataro JP, Guerrant RL. Etiology and epidemiology of persistent diarrhea in northeastern Brazil: a hospital-based, prospective, case-control study. J Pediatr Gastroenterol Nutr 1995;21:137-44.
41. Gonzalez R, Diaz C, Marino M, Cloralt R, Pequeneze M, Perez-Schael I. Age-specific prevalence of *Escherichia coli* with localized and aggregative adherence in Venezuelan infants with acute diarrhea. J Clin Microbiol 1997;35:1103-7.
42. Bouzari S, Jafari A, Farhoudi-Moghaddam AA, Shokouhi F, Parsi M. Adherence of non-enteropathogenic *Escherichia coli* to HeLa cells. J Med Microbiol 1994;40:95-7.
43. Henry FJ, Udoy AS, Wanke CA, Aziz KMA. Epidemiology of persistent diarrhea and etiologic agents in Mirzapur, Bangladesh. Acta Paediatr Suppl 1996;381:27-31.
44. Mathewson JJ, Oberhelman RA, DuPont HL, Javier de la Cabada F, Garibay EV. Enteroadherent *Escherichia coli* as a cause of diarrhea among children in Mexico. J Clin Microbiol 1987;25:1917-9.
45. Vial PA, Mathewson JJ, DuPont HL, Guers L, Levine MM. Comparison of two assay methods for patterns of adherence to HEp-2 cells of *Escherichia coli* from patients with diarrhea. J Clin Microbiol 1990;28:882-5.
46. Cravioto A, Gross RJ, Scotland SM, Rowe B. An adhesive factor found in strains of *Escherichia coli* belonging to the traditional infantile enteropathogenic serotypes. Current Microbiology 1979;3:95-9.
47. Baudry B, Savarino SJ, Vial P, Kaper JB, Levine MM. A sensitive and specific DNA probe to identify enteroaggregative *Escherichia coli*, a recently discovered diarrheal pathogen. J Infect Dis 1990;161:1249-51.
48. Kang G, Mathan MM, Mathan VI. Evaluation of a simplified HEp-2 cell adherence assay for *Escherichia coli* isolated from South Indian children with acute diarrhea and controls. J Clin Microbiol 1995;33:2204-5.
49. Schmidt H, Knop C, Franke S, Aleksic S, Heeseman J, Karch H. Development of PCR for screening of enteroaggregative *Escherichia coli*. J Clin Microbiol 1995;33:701-5.
50. Bhan MK, Khoshoo V, Sommerfelt H, Raj P, Sazawal S, Srivastava R. Enteroaggregative *Escherichia coli* and Salmonella associated with nondysenteric persistent diarrhea. Pediatr Infect Dis J 1989;8:499-502.
51. Bhatnagar S, Bhan MK, Sommerfelt H, Sazawal S, Kumar R, Saini S. Enteroaggregative *Escherichia coli* may be a new pathogen causing acute and persistent diarrhea. Scand J Infect Dis 1993;25:579-83.
52. Cobeljic M, Miljkovic-Selimovic B, Paunovic-Todosijevic D, Velickovic Z, Lepsanovic Z, Zec N, et al. Enteroaggregative *Escherichia coli* associated with an outbreak of diarrhoea in a neonatal nursery ward. Epidemiol Infect 1996;117:11-6.
53. Bhan MK, Bhandari N, Sazawal S, Clemens J, Raj P, Levine MM, Kaper JB. Descriptive epidemiology of persistent diarrhea among young children in rural northern India. Bull World Health Organ 1989;67:281-8.
54. Itoh Y, Nagano I, Kunishima M, Ezaki T. Laboratory investigation of enteroaggregative *Escherichia coli* O untypeable:H10 associated with a massive outbreak of gastrointestinal illness. J Clin Microbiol 1997;35:2546-50.
55. Smith HR, Scotland SM, Willshaw GA, Rowe B, Cravioto A, Eslava C. Isolates of *Escherichia coli* O44:H18 of diverse origin are enteroaggregative. J Infect Dis 1994;170:1610-3.
56. Smith HR, Cheasty T, Rowe B. Enteroaggregative *Escherichia coli* and outbreaks of gastroenteritis in UK. Lancet 1997;350:814-5.
57. Morais TB, Gomes TAT, Sigulem DM. Enteroaggregative *Escherichia coli* in infant feeding bottles. Lancet 1997;349:1448-9.
58. Huppertz HI, Rutkowski S, Aleksic S, Karch H. Acute and chronic diarrhoea and abdominal colic associated with enteroaggregative *Escherichia coli* in young children living in western Europe. Lancet 1997;349:1660-2.
59. Pai M, Kang G, Ramakrishna BS, Venkataraman A, Muliyil J. An epidemic of diarrhoea in south India caused by enteroaggregative *Escherichia coli*. Indian J Med Res 1997;106:7-12.
60. Checkley W, Gilman RH, Epstein LD. Asymptomatic and symptomatic cryptosporidiosis: their acute effect of weight gain in Peruvian children. Am J Epidemiol 1997;145:156-63.

61. Guerrant R, Schorling J, McAuliffe J, Souza MD. Diarrhea as a cause and effect of malnutrition: diarrhea prevents catch-up growth and malnutrition increases diarrhea frequency and duration. Am J Trop Med Hyg 1992;47:28-35.

62. Schorling J, McAuliffe J, Souza MD, Guerrant RL. Malnutrition is associated with increased diarrhea incidence and duration among children in an urban Brazilian slum. Int J Epidemiol 1990;19:728-35.
